# Biodegradation of Inosine and Guanosine by *Bacillus paranthracis* YD01

**DOI:** 10.3390/ijms241914462

**Published:** 2023-09-23

**Authors:** Xinyue Du, Yao Jiang, Yawen Sun, Xiaoyu Cao, Yu Zhang, Qianqian Xu, Hai Yan

**Affiliations:** School of Chemistry and Biological Engineering, University of Science and Technology Beijing, Beijing 100083, China; m202120900@xs.ustb.edu.cn (X.D.);

**Keywords:** inosine, guanosine, biodegradation, *Bacillus paranthracis* YD01, genome analysis

## Abstract

Both inosine and guanosine are precursors of uric acid that may cause the diseases of hyperuricemia and gout in humans. Here, a promising bacterial strain for efficiently biodegrading both inosine and guanosine was successfully isolated from a healthy human intestine and identified as *Bacillus paranthracis* YD01 with 16S rRNA analysis. An initial amount of 49.6 mg·L^−1^ of inosine or 49.9 mg·L^−1^ of guanosine was completely removed by YD01 within 12 h, which showed that YD01 had a strong ability to biodegrade inosine and guanosine. Furthermore, the initial amount of 49.2 mg·L^−1^ of inosine or 49.5 mg·L^−1^ of guanosine was totally catalyzed by the intracellular crude enzymes of YD01 within 6 h, and the initial inosine amount of 49.6 mg·L^−1^ or guanosine of 49.7 mg·L^−1^ was biodegraded by the extracellular crude enzymes of YD01 within 9 h. Illumina Hiseq sequencing and database gene annotation were used to elucidate the genomic characteristics of *B. paranthracis* YD01. Purine nucleoside phosphorylase, encoded by gene 1785, gene 3933, and gene 4403, was found in the KEEG database, which played a crucial role in the biodegradation of inosine and guanosine. The results of this study provide valuable insights into the mechanisms for biodegrading inosine and guanosine using *B. paranthracis* YD01.

## 1. Introduction

The intake of high-purine foods gradually significantly increases the incidence of metabolic diseases such as hyperuricemia [1,2], which is a condition in which the level of uric acid (UA) in the blood is higher than normal due to the excessive production or impaired excretion of UA in the body under normal dietary conditions [3,4,5]. Hyperuricemia is influenced by many factors and has a certain correlation with genetics, gender, age, race, environment, diet, and lifestyle [6,7]. However, one of the most important causes of hyperuricemia is a disorder of purine metabolism [8,9,10]. Excessive inosine or guanosine intake will interfere with the metabolism of purine nucleoside in the body, resulting in an imbalance of purine nucleoside metabolism in the body and leading to abnormal accumulation and high levels of UA, causing hyperuricemia and gout, which seriously affect people’s health and quality of life [11,12,13].

**Figure 1:**
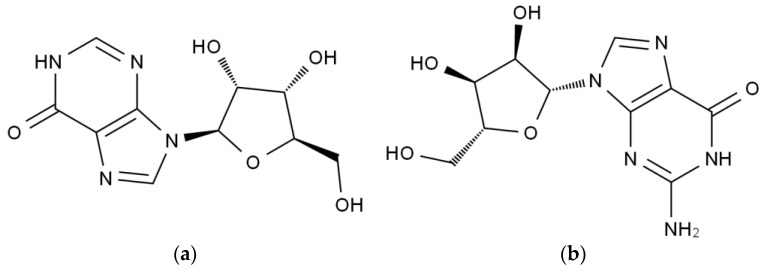
(**a**) Chemical structure of inosine. (**b**) Chemical structure of guanosine.

Inosine is a nucleoside compound formed by the combination of hypoxanthine and ribose, also known as hypoxanthine nucleoside, with the molecular formula of C_10_H_12_N_4_O_5_ (Figure 1a) [14,15]. Guanosine is an organic compound with the molecular formula of C_10_H_13_N_5_O_5_, a molecular weight of 283 Daltons, and a density of 2.25 g·cm^−3^ (Figure 1b) [16,17]. Both inosine and guanosine are catalyzed by many different enzymes and are ultimately converted to UA [18,19]. Firstly, inosine and guanosine are converted by purine nucleoside phosphorylase (PNP) to hypoxanthine and guanine, respectively. Then, hypoxanthine and guanine are converted to xanthine by xanthine oxidase (XO) and guanine deaminase, respectively. Finally, xanthine is further oxidized by XO to UA, which is the final biodegradation product of purine nucleotides [20,21].

The treatment of hyperuricemia mainly includes the following aspects. Lifestyle changes and the strict control of the intake of purine-rich foods such as meat, seafood, and animal organs can alleviate the symptoms of hyperuricemia [22,23,24]. Furthermore, some drugs such as allopurinol, benzbromarone, pegalogenase, and losartan can also participate in the treatment of hyperuricemia by inhibiting the synthesis of UA or promoting the degradation and excretion of UA [25,26,27,28]. However, the above-mentioned drugs may cause various degrees of side effects and cannot achieve the expected effect of clinical treatment [29]. Therefore, it is necessary to find and develop the bacteria from human microorganisms for the prevention and cure of hyperuricemia.

Biodegradation is an effective method for the removal of purine nucleosides due to its low cost and low disruption of the microecological balance of the human gut [30]. It has been reported that some probiotics can maintain intestinal flora homeostasis, promote purine metabolism [31], and reduce UA levels in the body by biodegrading inosine and guanosine [32]. Lactic acid bacteria, as an inherent beneficial flora in the gut, were found to absorb or hydrolyze purine nucleosides in food and regulate the intestinal flora [33,34]. The microbial degradation of inosine and guanosine is achieved by screening bacteria with specific physiological activities and using these beneficial bacteria to prepare microbial preparations that have beneficial effects on the body and improve the intestinal microbial balance [35]. A study has shown that *Lactobacillus gasseri* PA-3 can degrade extracellular nucleosides, absorb nucleosides into the cell, reduce intestinal absorption of nucleosides, and then achieve the effect of reducing blood UA [36]. *Lactobacillus brevis* DM9218, isolated by Wang et al., could prevent liver injury induced by high fructose and reduce serum UA levels by degrading inosine [37]. In addition, Kuo et al. confirmed that *Lactobacillus reuteri* TSR332 and *Lactobacillus fermentum* TSF331 could stabilize the serum uric acid level in rats and prevent hyperuricemia [38]. However, no report was found on the biodegradation of inosine and guanosine using *Bacillus paranthracis* from healthy human intestines.

In this study, a promising bacterial strain of *B. paranthracis* YD01, which can biodegrade both inosine and guanosine, was successfully isolated from the intestines of healthy humans. Both the cells of *B. paranthracis* YD01 and its intracellular and extracellular crude enzymes were found to have a strong ability to biodegrade both inosine and guanosine efficiently. The genome sequencing of *B. paranthracis* YD01 indicated that the purine nucleoside phosphorylase, encoded by gene 1785, gene 3933, and gene 4403 found in the KEEG database, played a key crucial role in the biodegradation of inosine and guanosine. This can provide an important theoretical basis for removing both inosine and guanosine using *B. paranthracis* YD01.

## 2. Results and Discussion

### 2.1. Isolation and Identification of Strain

**Figure 2:**
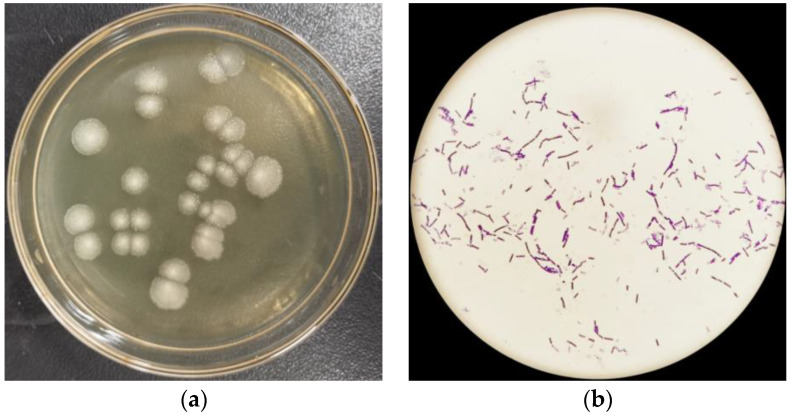
(**a**) Colony morphology of YD01. (**b**) Microscopic morphology of YD01 (Scale bar, 1000:1).

When the initial concentration of inosine or guanosine as the sole carbon source was at 5.0 g·L^−1^ in a culture medium, a bacterial strain (YD01) was successfully isolated from the intestines of healthy humans. YD01 formed white circular colonies on the solid medium (Figure 2a), and the cells of YD01 were Gram-positive, rod-shaped, and spore-bearing, as observed under a microscope (Figure 2b).

**Figure 3:**
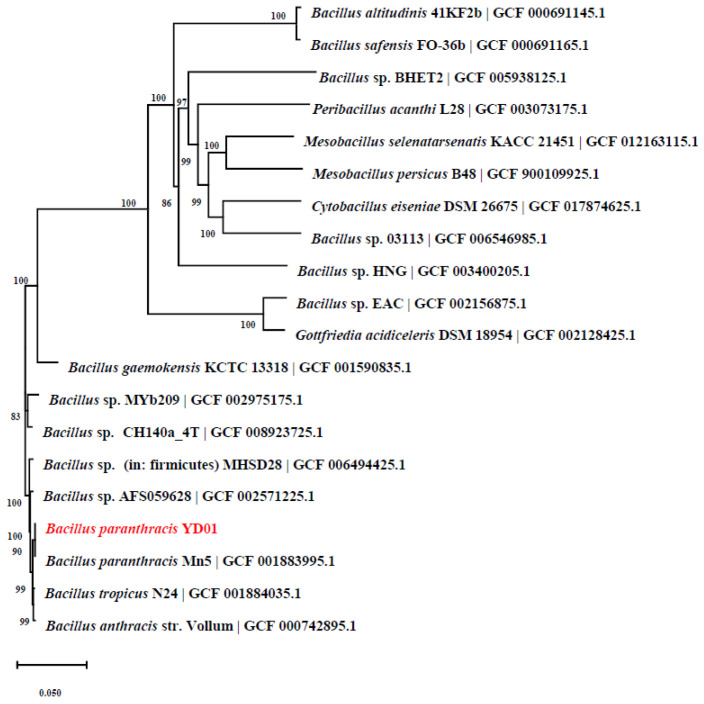
Phylogenetic tree of *Bacillus paranthracis* YD01 based on 16S rRNA sequence. Phylogenetic relationships between species were assessed at the genomic level using average nucleotide identity (ANI) analysis. Throughout the analysis, the identity between strain YD01 and *B. paranthracis* is 100%. The red font represented the strain screened in this study.

The nucleic acid sequence of YD01 was amplified and sequenced to determine its phylogenetic placement [39]. The 16S rRNA sequence analysis showed that YD01 was closely related to *B. paranthracis* and belonged to the same evolutionary branch (Figure 3). Based on the morphological and physiological characteristics and the phylogenetic analysis of 16S rRNA sequences, YD01 was identified as *B. paranthracis* YD01. The draft genome sequence of *B. paranthracis* DB-4 was reported previously [40]. *B. paranthracis* ICIS-279 isolated from the human intestine was also reported to have tumor necrosis factor α (TNF-)-inhibitory activity [41]. In addition, the genome analysis of *B. paranthracis* strain MHSD3 indicated that it is an excellent potential probiotic [42]. Up to now, no report on the biodegradation of inosine and guanosine using *B. paranthracis* has been found.

### 2.2. Biodegradation of Inosine and Guanosine Using B. paranthracis YD01

**Figure 4:**
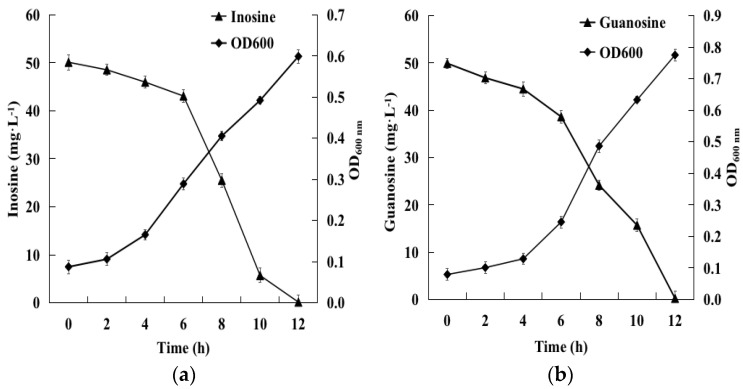
Growth and biodegradation kinetics of inosine (**a**) and guanosine (**b**) by YD01.

The initial inosine amount of 49.6 mg·L^−1^ was completely removed within 12 h by *B. paranthracis* YD01 (Figure 4a), and the initial guanosine amount of 49.9 mg·L^−1^ was completely removed within 12 h by *B. paranthracis* YD01 (Figure 4b), indicating that the isolated strain had good biodegradability for both inosine and guanosine. At present, inosine and guanosine have been shown to be well-biodegradable by *Lactobacillus reuteri* and *Lactobacillus fermentum*. Kuo et al. confirmed that *Lactobacillus reuteri* TSR332 and *Lactobacillus fermentum* TSF331 displayed a significantly strong removal of inosine (90%; *p* = 0.00003) and guanosine (78%; *p* = 0.00012) within 30 min in vitro. Compared with other purine-biodegrading strains reported [38], YD01 is a promising bacterial strain for the efficient biodegradation of inosine and guanosine.

### 2.3. Biodegradation of Inosine and Guanosine by Intracellular and Extracellular Crude Enzymes

**Figure 5:**
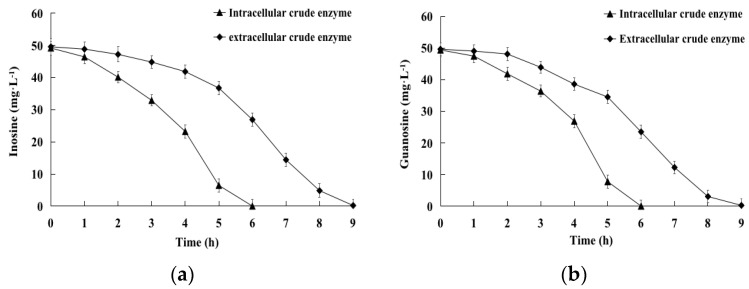
(**a**) Kinetics of inosine catalyzed by intracellular and extracellular crude enzymes. (**b**) Kinetics of guanosine catalyzed by intracellular and extracellular crude enzymes.

The initial inosine amount of 49.2 mg·L^−1^ was completely biodegraded within 6 h or 9 h by intracellular or extracellular crude enzymes with a protein concentration of 0.3 g·L^−1^ or 1.9 g·L^−1^ (Figure 5a). The initial guanosine amount of 49.5 mg·L^−1^ was completely biodegraded within 6 h or 9 h by intracellular or extracellular crude enzymes with a protein concentration of 0.3 g·L^−1^ or 2.0 g·L^−1^ (Figure 5b). Both intracellular and extracellular crude enzymes showed good biodegradation rates, which is of great significance for the effective control of inosine and guanosine.

### 2.4. Genome Assembly and Prediction of YD01

The short-sequence assembly software SOAPdenovo2 was used to splice multiple K-mer parameters of the second-generation sequenced optimized sequences to obtain the optimal contig assembly result, and then the reads were aligned to the contig. The total number of scaffolds for each genome was 50, the total length of all scaffolds was 5,488,402 bp, and the maximum length of scaffolds was 1,583,563 bp. The total number of contigs for each genome was 59, the total length of all contigs was 5,488,159 bp, and the maximum contig length was 1,299,460 bp (Table 1). The draft genome sequence of *B. paranthracis* DB-4 and the genome sequence of *B. paranthracis* ICIS-279 were reported previously. The assembly of DB-4 revealed 57 contigs covering a total of 5,424,208 bp, with an N_50_ value of 498,305 bp, a GC content of 35.2%, and a coding ratio of 84.0% [40]. Additionally, the genome size of ICIS-279 is 5,180,499 bp, with a GC content of 35.4%, and annotation revealed 5168 coding sequences, including 5168 proteins and 43 rRNA, 102 tRNA, and 5 ncRNA genes [41].

The coding sequence (CDS) in the genome of YD01 was predicted by Glimmer software (http://ccb.jhu.edu/software/glimmer/index.shtml, accessed on 5 August 2023). A total of 5600 protein-coding genes were predicted, with a total gene length of 4,654,437 bp, an average gene length of 831.15 bp, and a gene density of 1.02, and the chromosomal genome coding genes accounted for 84.80% of the total genes (Table 2).

**Figure 6:**
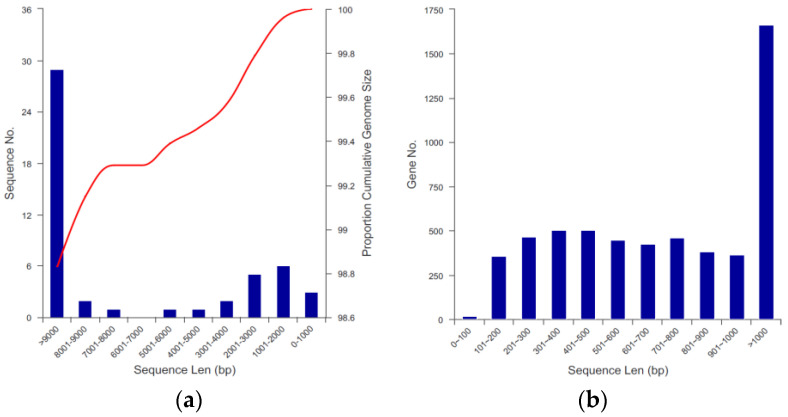
(**a**) Assembly distribution of genomes. The red curve corresponded to genome size accumulation. (**b**) Length distribution of coding genes.

Most of the sequence lengths of *B. paranthracis* YD01 were between 1000 bp and 9000 bp, among which the number of sequence lengths above 9000 bp was the largest and the number of sequence lengths between 6001 bp and 7000 bp was the smallest. The horizontal coordinate was the sequence length range, the left vertical coordinate was the number of scaffolds (or contigs), and the right vertical coordinate was the proportion of accumulated genome size (accumulated from left to right). The bars represent the number of scaffolds (or contigs) and the curves represent the accumulation of genome sizes (Figure 6a). Most of the coding gene lengths of *B. paranthracis* YD01 ranged from 100 bp to 1000 bp, among which the number of coding gene lengths above 1000 bp was the largest and the number of coding gene lengths below 100 bp was the smallest. The horizontal coordinate is the length range of the genes, and the vertical coordinate is the number of genes (Figure 6b).

### 2.5. Genomic Circle Map Analysis and Gene Annotation of YD01

**Figure 7:**
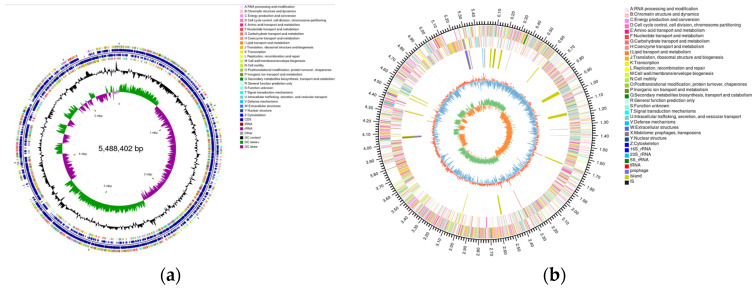
(**a**) CGView genome circle map of YD01. (**b**) Circos genome circle map of YD01.

The CGView genome circle map comprehensively displays the characteristics of the genome. From outside to inside, the first and fourth circles are CDSs on the positive and negative chains, respectively, and different colors indicate different COG functional classifications. The second and third circles are CDSs, tRNA, and rRNA on the positive and negative chains, respectively. The fifth circle is the GC content. The outward part indicates that the GC content of this region was higher than the average GC content of the whole genome; the inward part indicates that the GC content of this region was lower than the average GC content of the whole genome; and the higher the peak value, the greater the difference between the GC content and the average GC content. The sixth circle is the GC skew value. The innermost circle indicates the size of the genome (Figure 7a). The Circos genome map added new information circles, such as ncRNA, Prophage, GI, and IS, and the information corresponding to the outer circle to the inner circle includes the genome size identifier, the gene information on the positive chain, the negative chain, ncRNA, GC content, and GC skew (Figure 7b).

The annotation results of each database were summarized at the gene level to realize multidimensional data mining of genes. Based on protein sequence alignment, gene sequences were compared with each database to obtain the corresponding functional annotation information. There were 5594 CDSs of YD01 annotated in the Non-Redundant Protein Database (NR), 4043 CDSs annotated in the Swiss-prot Database, 4613 CDSs annotated in the Pfam Database, 4161 CDSs annotated in the Clusters of Orthologous Groups of Proteins Database (COG), 3824 CDSs annotated in the Gene Ontology Database (GO), and 2691 CDSs annotated in the Kyoto Encyclopedia of Genes and Genomes Database (KEGG) (Table 3).

**Figure 8:**
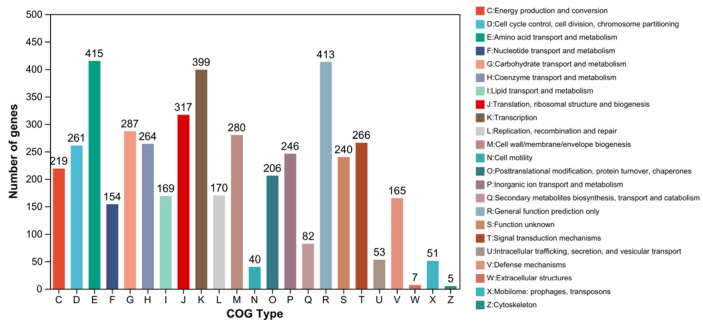
COG gene annotation of *B. paranthracis* YD01.

There were 4161 genes in the genome of *B. paranthracis* YD01 assigned to 23 functional categories in the COG database, and the genes annotated by COG accounted for 74.30% of all genes. There were 413 gene annotations associated only with general function prediction (R), accounting for 8.95%. There were 415 gene annotations related to amino acid transport and metabolism (E), accounting for 8.99%. There were 399 gene annotations related to transcriptional function (K), accounting for 8.65%. There were 317 gene annotations related to translation, ribosome structure, and biogenesis (J), accounting for 6.87% (Figure 8).

**Figure 9:**
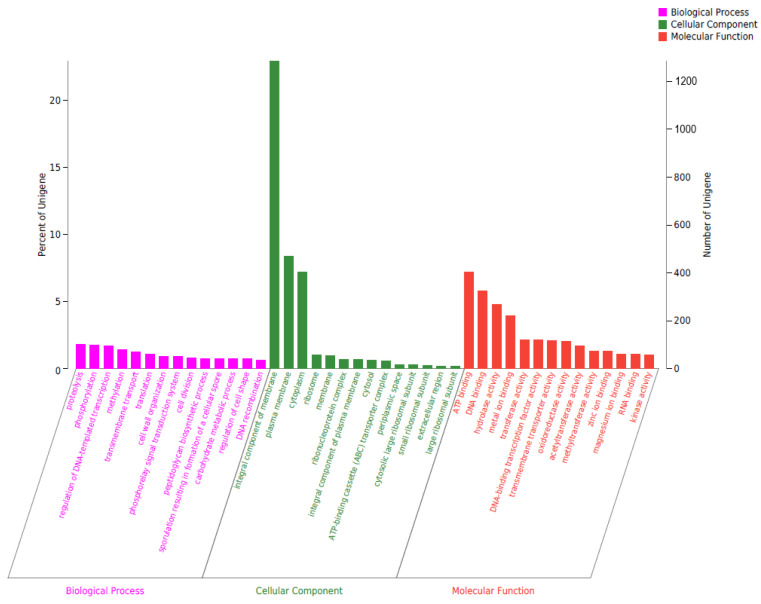
GO gene annotation of *B. paranthracis* YD01.

A total of 3824 genes of *B. paranthracis* YD01 were annotated in the GO database. The horizontal coordinate represents the three branches of GO and the further level-2 classification, and the vertical coordinate represents the relative proportion of genes (Figure 9). There were 1787 genes related to biological processes, accounting for 46.73%. There were 1990 genes related to the cellular component, accounting for 52.04%. There were 2929 genes related to molecular function, accounting for 76.60%. Biological process included proteolysis, phosphorylation, regulation of DNA-templated transcription, methylation, transmembrane transport, and other function-related genes. The cellular component included the integral components of membrane, plasma membrane, cytoplasm, ribosome, membrane, ribonucleoprotein complex, and other function-related genes. Molecular function included ATP binding, DNA binding, hydrolase activity, metal ion binding, transferase activity, and other function-related genes.

**Figure 10:**
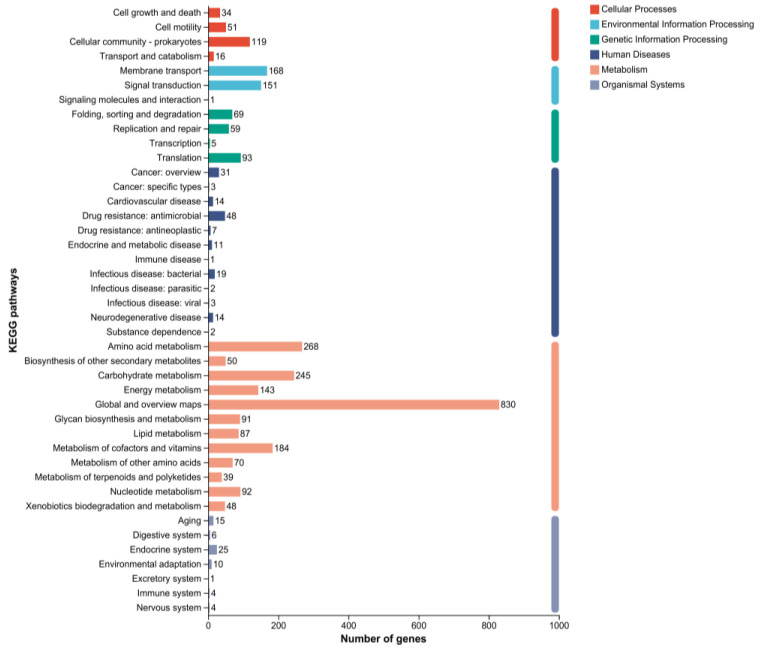
KEGG gene annotation of *B. paranthracis* YD01.

A total of 2691 genes of *B. paranthracis* YD01 were annotated in the KEGG database. The horizontal coordinate represents the level-2 classification of the KEGG pathway, and the vertical coordinate represents the number of genes annotated under this classification. The colors of the columns represent the level-1 classification of the KEGG pathway. The right column represents the number of genes in different level-1 categories. Since the same gene was annotated in multiple level-2 classes, the number of genes in the level-1 class was calculated to eliminate redundancy (Figure 10). In the metabolic classification, amino acid metabolism, carbohydrate metabolism, metabolism of cofactors and vitamins, energy metabolism, nucleotide metabolism, glycan biosynthesis and metabolism, biosynthesis of other secondary metabolites, xenobiotics biodegradation and metabolism, lipid metabolism, metabolism of terpenoids and polyketides, and other related metabolic pathways were annotated. In addition, the KEGG database also annotated cellular processes, environmental information processing, genetic information processing, human diseases, and the organismal system.

**Figure 11:**
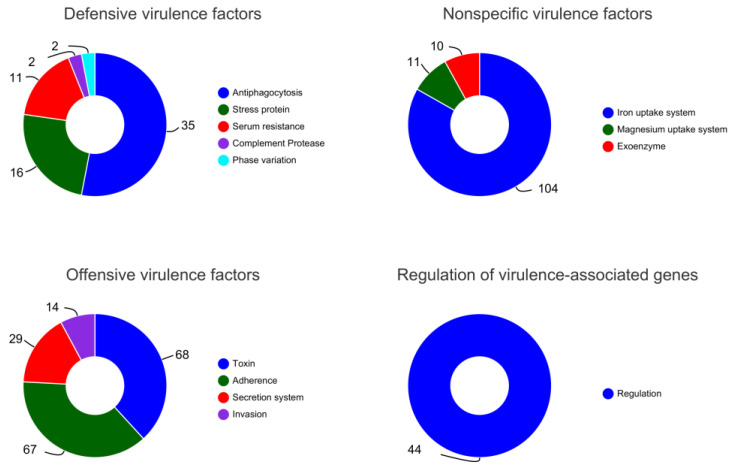
VFDB gene annotation of *B. paranthracis* YD01.

A total of 513 virulence genes related to *B. paranthracis* YD01 were annotated by the VFDB database comparison. Classification was conducted using virulence factors, including defensive virulence factors, nonspecific virulence factors, offensive virulence factors, and the regulation of virulence-associated genes. According to the secondary classification of virulence factors, it mainly included 13 categories, including iron uptake system, toxin, adherence, regulation, antiphagocytosis, and secretion system. The results showed that *B. paranthracis* YD01 had the largest number of virulence factors and related genes belonging to the iron uptake system, with a total of 104 virulence genes. The text above each pie chart is the primary classification of virulence factors, and the text to the right of the pie chart is the secondary classification of virulence factors. Different colors of the pie charts represent different secondary classifications, and their areas represent the relative proportion of genes in that classification (Figure 11). In contrast to the previously reported isolation of *B. paranthracis* BCCL01 from the book page surface [43], YD01 did not annotate related virulence factors such as inhibitor A metalloprotease (*inhA*) and the cluster for non-hemolytic enterotoxin (*nheA*/*B*/*C*). Therefore, *B. paranthracis* YD01 does not have pathogenicity similar to these virulence factors.

### 2.6. Gene Annotation of Biodegrading Enzyme of YD01

**Figure 12:**
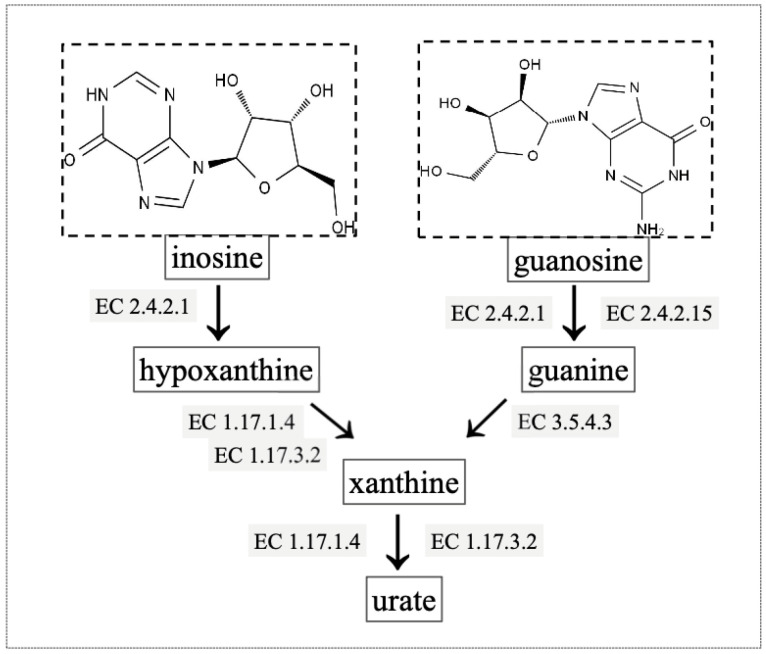
Metabolic pathway for the biodegradation of inosine and guanosine using *B. paranthracis* YD01. [EC:2.4.2.1], purine nucleoside phosphorylase; [EC:2.4.2.15], guanosine phosphorylase; [EC:1.17.1.4], xanthine dehydrogenase; [EC:1.17.3.2], xanthine oxidase; [EC: 3.5.4.3], guanine deaminase.

Purine nucleoside phosphorylase, encoded by gene 1785, gene 3933, and gene 4403, was found in the KEEG database, which was involved in the biodegradation of inosine and guanosine. Firstly, inosine and guanosine are converted by purine nucleoside phosphorylase (PNP) to hypoxanthine and guanine, respectively. Then, both hypoxanthine and guanine were converted to xanthine by xanthine oxidase and guanine deaminase, respectively. Finally, xanthine was converted to urate with the participation of xanthine dehydrogenase or oxidase (Figure 12). Several genes that encode the biodegradation of inosine and guanosine were conserved. For instance, the purine nucleoside phosphorylase genes *deoD* and *yfiH* were conserved throughout evolution, and these genes were also found in the *B. paranthracis*-type strain Bt C4. However, there were also related degradation genes that were unique to YD01, such as the gene *punA*, which was not found in this type of strain or other strains of *B. paranthracis*.

In addition to gene 1785, gene 3933, and gene 4403, we identified other genes encoding enzymes associated with purine metabolic pathways in humans (Table 4). These enzymes biodegrade UA precursors such as inosine, guanosine, xanthine, hypoxanthine, and adenine. In a previous report, whole-genome sequencing of *L. brevis* DM9218 was performed by Haina Wang et al., who identified the gene named ORF00084 and verified inosine hydrolytic ability [37]. The gene product was an inosine hydrolase that reduced UA production by biodegrading inosine—the main precursor of UA synthesis. Now, the biodegradation of xanthine, hypoxanthine, and adenine by the cells and crude enzymes of YD01 is being further investigated.

## 3. Materials and Methods

### 3.1. Materials

The strain used in this study was isolated from the intestines of healthy humans, with inosine or guanosine as the sole carbon and energy source. The intestinal flora samples were purchased from Beijing Fumat Biotechnology Co., Ltd. (Beijing, China). Standard 99% pure inosine was purchased from Macklin Biochemical Co., Ltd. (Shanghai, China). Standard 99% pure guanosine was purchased from Yuanye Bio-Technology Co., Ltd. (Shanghai, China). All other chemicals used were of analytical or chromatographic grade.

### 3.2. Culture Medium and Conditions

The basal liquid medium for isolation and culture of strain YD01 consisted of 0.5 g of NH_4_Cl, 0.5 g of KH_2_PO_4_, 0.5 g of Na_2_CO_3_, 0.1 g of MgSO_4_·7H_2_O, 0.5 g of peptone, 0.5 g of yeast powder, and 0.1% of Tween-80 in 1000 mL distilled water. The initial pH of the medium was adjusted to 7.0 with 1.0 mol·L^−1^ HCl or NaOH. Inosine or guanosine with different initial concentrations were added to the medium as the sole carbon and energy sources. The medium and all experimental equipment were sterilized at 121 °C for 20 min. The YD01 was inoculated into the sterilized culture medium and grown in a 100 mL flask containing 50 mL of liquid medium. The culture condition was at the temperature of 38 °C with a shaking rate of 200 rpm. The optical density at a wavelength of 600 nm (OD_600_) was measured, which represented the growth of YD01. The culture broth of 1.0 mL was taken and centrifuged at 12,000 rpm for 15 min, the supernatant was diluted and filtered, and the concentration of inosine or guanosine was determined directly by HPLC.

### 3.3. Isolation of Inosine and Guanosine Biodegrading Strain

Human intestinal flora samples were resuspended in sterile saline. The 1% solution was inoculated into 50 mL of liquid medium and incubated in an incubator shaker at 38 °C at 200 rpm for 5 d. Every 3–5 d, 1.0 mL of the cultures was subcultured into a medium with the same culture conditions each time. The concentration of inosine and guanosine was 5.0 g·L^−1^. After 5–6 weeks, the last continuously diluted culture was plated on LB agar using the plating method and incubated at 38 °C for 2 d. Individual colonies grown on LB agar plates were selected and inoculated into a modified medium containing inosine or guanosine to test for biodegradability, which was repeated several times until pure strains were isolated.

### 3.4. Identification and Genome Sequencing of YD01

The morphology of YD01 was observed by microscopy. The YD01 was inoculated on LB medium and incubated at 38 °C with a shaking speed of 200 rpm for 48 h. The culture solution of YD01 was prepared, and bacterial precipitates were collected by centrifugation at 12,000 rpm at 4 °C for 10 min and sent to Majorbio Bio-pharm Technology Co., Ltd. (Shanghai, China) for draft genome sequencing [44]. The bacterial genome was sequenced using de novo sequencing technology, and the genome sequence was assembled from scratch using bioinformatics. PCR was performed using a pair of universal primers: 27F (5′-GAGTTTTGATCCTGTCTCAGA-3′) and 1492R (5′-GGTACCTTGTTACGACTT-3′). The PCR conditions were as follows: pre-denaturation at 95 °C for 5 min; denaturation at 95 °C for 30 s; annealing at 56 °C for 30 s, extension at 72 °C for 90 s, 25 cycles; and extension at 72 °C for 10 min. The 16S rRNA sequences of selected strains were analyzed by BLAST through GenBank and the Ribosomal Database Project. Based on the 16S rRNA gene sequence, the MEGA11 neighbor-joining method was used to construct the phylogenetic tree.

### 3.5. Preparation of Intracellular and Extracellular Crude Enzymes of YD01

The YD01 was inoculated into 50 mL of sterilized LB medium and incubated at 38 °C with shaking at 200 rpm for 48 h. The YD01 culture was centrifuged at 15,000 rpm for 10 min. The sediment of YD01 cells was washed several times, resuspended with sterilized PBS (pH 7.0), and disrupted by ultrasound at 300 W for 20 min (Scientz JY92- IID, Ningbo, China). The intracellular and extracellular crude enzymes were obtained by centrifugation at 15,000 rpm for 10 min at 4 °C, and the protein concentration was determined at 280 nm. Then, 4% crude enzymes were added to sterilized PBS (pH 7.0) and inoculated into a medium containing inosine and guanosine concentrations of 50 mg·L^−1^, respectively. The reaction was performed at 38 °C with a shaking speed of 200 rpm for 12 h. The 1.0 mL of culture was taken at 0, 1, 2, 3, 4, 5, 6, 7, 8, and 9 h to measure the concentration of inosine and guanosine. The content of intracellular and extracellular crude enzyme protein was determined by ultraviolet spectrophotometry, and the standard curve of absorbance and protein concentration was plotted. The correlation coefficient of the regression equation R^2^ = 0.9996 showed a good correlation. The absorbance value corresponded to the protein concentration in the standard working solution by linear fitting, and the crude enzyme content could be calculated by combining the standard curve. Then, the biodegradation of inosine and guanosine by intracellular and extracellular crude enzymes could be obtained.

### 3.6. Analysis of Inosine and Guanosine by HPLC

Inosine and guanosine were measured by HPLC (Shimadzu LC-20AT, Tokyo, Japan). Samples of 1.0 mL were diluted with sterile water and centrifuged at 12,000 rpm for 10 min (Sigma 1–14, Berlin, Germany). The supernatant was passed through an aqueous microporous membrane (0.22 μm) and used for detection. Then, 20 µL solution was extracted and analyzed by HPLC (InertSustain C18 (4.6 × 250 mm, 5 μm); UV detection wavelength of inosine: 248 nm; UV detection wavelength of guanosine: 253 nm; mobile phase: 0.2% acetic acid aqueous solution: methanol (10:90); flow rate: 1 mL·min^−1^; column temperature: 35 °C). The standard curves of inosine and guanosine were determined by HPLC. The correlation coefficient of the inosine standard curve regression equation was R^2^ = 0.9999, and the correlation coefficient of the guanosine standard curve regression equation was R^2^ = 0.9999; both had a good correlation. The peak area corresponded to the concentration of inosine and guanosine in the standard working solution by linear fitting. Combined with the standard curve, the content of inosine and guanosine in the bacterial solution sample could be calculated, and then the bacterial biodegradation rate of inosine and guanosine could be obtained.

## 4. Conclusions

*B. paranthracis* YD01, an efficient bacterial strain for biodegrading inosine and guanosine, was successfully isolated from a healthy human intestine. At initial pH 7.0 and 38 °C, YD01 completely biodegraded initial inosine and guanosine amounts of 49.6 mg·L^−1^ and 49.9 mg·L^−1^ within 12 h. Moreover, the initial inosine amount of 49.2 mg·L^−1^ and guanosine amount of 49.5 mg·L^−1^ could be catalyzed by intracellular crude enzymes of YD01 within 6 h, and 49.6 mg·L^−1^ of inosine and 49.7 mg·L^−1^ of guanosine could be catalyzed by extracellular crude enzymes of YD01 within 9 h. These findings demonstrate YD01′s remarkable capacity for inosine and guanosine biodegradation. Importantly, genomic analysis revealed the presence of genes 1785, 3933, and 4403, encoding purine nucleoside phosphorylase, which was involved in the biodegradation of inosine and guanosine. Particularly, inosine and guanosine were converted to xanthine during purine metabolism, and xanthine was finally converted to urate with the participation of xanthine dehydrogenase or oxidase. Additionally, our study revealed five enzymes and thirteen genes encoding the biodegradation of other purines such as xanthine, hypoxanthine, and adenine. These findings reveal the metabolic pathway in the biodegradation of inosine and guanosine by YD01 and open a new avenue for future research on the regulation of purine nucleoside metabolism. We expect that these results will provide valuable insights into a theoretical basis for the adjuvant therapy of hyperuricemia and gout.

## Figures and Tables

**Table 1:** Statistical results of the genome assembly.

Attribute	Value
Total Scaf No.	50
Total Bases in Scaf (bp)	54,88,402
Largest Scaf Len (bp)	1,583,563
Total Ctg No.	66
Total Bases in Ctg (bp)	5,488,159
Largest Ctg Len (bp)	1,299,460

**Table 2:** Prediction results for coding genes.

Attribute	Value
Gene No.	5600
Gene Total Len (bp)	4,654,437
Gene Average Len (bp)	831.15
Gene Density	1.02
Gene Len/Genome (%)	84.80

**Table 3:** Statistical results of gene annotation.

Database	Gene No.
NR	5594
Swiss-Prot	4043
Pfam	4613
COG	4161
GO	3824
KEGG	2691

**Table 4:** Genes and corresponding enzymes involved in the biodegradation of purines in YD01.

Gene ID	Location	Enzyme
gene0825, gene1511, gene5057, gene5657, gene5546, gene5703	Scaffold1, Scaffold1, Scaffold15, Scaffold33, Scaffold23, Scaffold47	purine nucleosidase [EC:3.2.2.1]
gene1091, gene4107, gene4389	Scaffold1, Scaffold7, Scaffold8	5′-nucleotidase [EC:3.1.3.5]
gene2567, gene5357	Scaffold3, Scaffold19	hypoxanthine phosphoribosyltransferase [EC:2.4.2.8]
gene1682	Scaffold2	xanthine phosphoribosyltransferase [EC:2.4.2.22]
gene3002	Scaffold3	adenine phosphoribosyltransferase [EC:2.4.2.7]

## Data Availability

Not applicable.
